# Heat Transfer Enhancement in Industrial Heat Exchangers Using Graphene Oxide Nanofluids

**DOI:** 10.1021/acsomega.4c02581

**Published:** 2024-05-24

**Authors:** Omid Khouri, Hamid Reza Goshayeshi, Seyed Borhan Mousavi, Shamin Hosseini Nami, Saeed Zeinali Heris

**Affiliations:** † School of Safety Science & Engineering, Xi’an University of Science and Technology, 58, Yanta Mid. Rd., Xi’an, Shaanxi 710054, China; ‡ Department of Mechanical Engineering, Mashhad Branch, Islamic Azad University, Mashhad 19585-466, Iran; § J. Mike Walker ‘66 Mechanical Engineering Department, Texas A&M University, College Station, Texas 77843, United States; ∥ School of Chemical, Biological and Materials Engineering, The University of Oklahoma, Norman, Oklahoma 73019, United States; ⊥ Faculty of Chemical and Petroleum Engineering, University of Tabriz, Tabriz 51666-16471, Iran

## Abstract

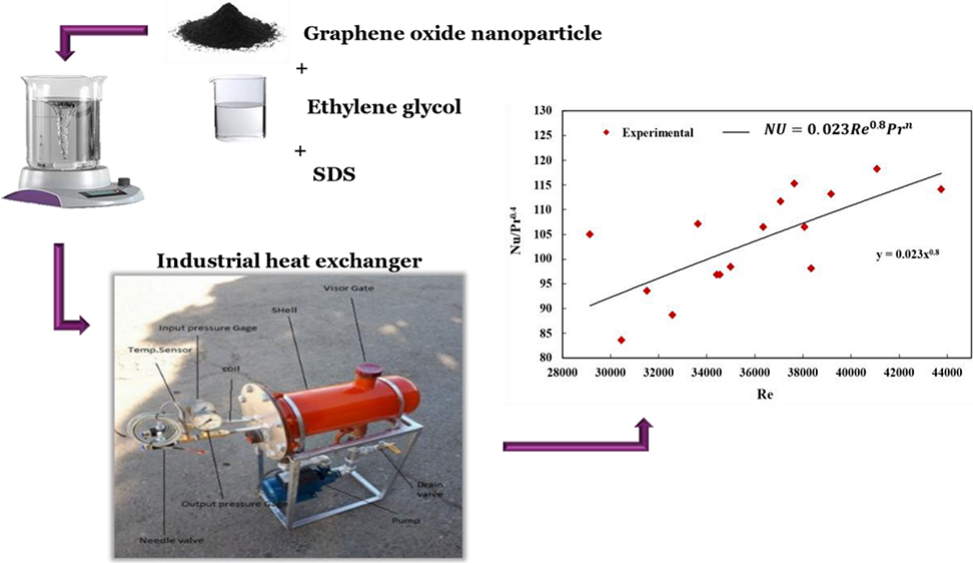

In this study, the heat transfer characteristics within the heat exchanger using water-based GO nanofluids were comprehensively assessed. An apparatus was constructed by scaling down an industrial heat exchanger. The nanofluid’s thermal conductivity, specific heat capacity, viscosity, density, Prandtl number, and Nusselt number were examined at varying temperatures and GO nanoparticle concentrations. The results revealed that the thermal conductivity of the nanofluid increased with both temperature and nanoparticle concentration, reaching a peak value of 0.380 W m^–1^ K^–1^ at 85 °C and 0.1 wt %, leading to enhanced heat transfer rates through conduction and convection mechanisms. The specific heat capacity increased with temperature but decreased with higher GO nanoparticle contents with a maximum value of 3403.821 J kg^–1^ K^–1^ recorded at 40 °C and 0.01 wt %. The viscosity of the nanofluid increased with higher concentrations of GO nanoparticles, and the minimum value of 0.83 mPa s was observed at 85 °C and 0.01 wt %. The Prandtl number decreased with the temperature but increased with increasing GO nanoparticle concentration, suggesting a transition from convective to conductive heat transfer. A newly derived correlation equation for the Nusselt number, *Nu* = 0.0059(1 + 7.62ϕ^0.6886^)*Pe*^0.001^*Re*^0.9238^*Pr*^0.4^, allows predicting heat transfer enhancement in nanofluids. The findings emphasize the potential of nanofluids for improving heat exchanger performance and offer valuable insights into optimizing nanofluid applications in thermal systems.

## 1 Introduction

One of the largest sources of fossil fuels is natural gas (NG), which must undergo high-pressure conveyance to counter pressure losses incurred during transportation from the refinery to consumption points. In contrast, domestic and industrial applications require lower pressure levels; hence, city gate stations (CGSs) are strategically installed at consumption points to reduce the gas pressure.^1−4^ Sudden pressure drops can cause a significant decrease in the temperature; therefore, to prevent natural gas from hydrating in CGSs, heat exchangers are employed to heat the gas prior to the pressure reduction stage. Conventional CGSs use indirect water bath heat exchangers, which burn a substantial amount of NG as a fuel for preheating. Given the prominent role of NG as a source and its consequential impact on greenhouse gas emissions, increasing fuel consumption, escalating energy prices, and imposing environmental standards, it becomes imperative to undertake substantial modifications in the design of thermal heat exchangers to significantly mitigate their environmental impacts.^5,6^ Heat exchangers conventionally utilize fluids with relatively low thermal conductivity, resulting in limited heat transfer rates and necessitating large-sized exchangers with suboptimal efficiency. However, the integration of fluids exhibiting enhanced thermal conductivity, compared with traditional counterparts, offers the potential for highly efficient and compact heat exchangers. Consequently, implementing nanofluids has emerged as a promising passive approach to exploiting this phenomenon.^7−9^

A nanofluid is described as a stable dispersion of nanoparticles in a liquid medium, where the nanoparticles exhibit sizes ranging from 1 to 100 nm and are suspended within base fluids, e.g., water or ethylene glycol.^10^ The utilization of nanofluids has garnered extensive use owing to their attribution to the enhancement of heat transfer and various thermophysical characteristics such as viscosity,^11^ flash point,^12,13^ thermal conductivity,^14,15^ heat and mass transfer coefficients, and cooling rate.^16^ Improvements in thermophysical properties depend on the stability of nanofluid, which is governed by several parameters, including the base fluid type, nanoparticle characteristics (type, size, and morphology), surfactant selection, and the nanofluid synthesis method.^17−19^ The extensive investigations conducted by Heris et al.’s research team^20,21^ revealed that incorporating nanofluids instead of base fluids yielded considerable enhancements in the utilized fluid’s thermophysical and heat transfer characteristics. The improvements were manifested in various parameters, such as velocity, pressure drop, cooling capacity, and thermal coefficients, all emanating from the extraordinary attributes of nanoparticles due to their pronounced features, such as substantial surface area, diminutive dimensions, and exceptional heat capacity. An extensive array of nanoparticles has been subjected to scrutiny with the specific aim of enhancing the heat exchange performance of different base fluids. Prominently studied nanoparticles are TiO_2_,^22^ Al_2_O_3_,^23^ MWCNT,^24^ ZnO,^25,26^ CuO,^27^ graphene oxide (GO),^28^ etc. Balaga et al.^29^ assessed the heat transfer properties of nanofluids with varying weight percentages of nanoparticles (0.01, 0.02, and 0.03%), with the functionalized multiwalled carbon nanotubes (f-MWCNTs) accounting for 60% by weight and Fe_2_O_3_ nanoparticles constituting 40% by weight. The outcomes of their study demonstrated that a higher nanoparticle concentration engenders an increase in both heat transfer and pressure drop; moreover, the observed enhancement in heat transfer ranged from 21.66 to 31.66%. Hemmat Esfe et al.^30^ conducted a comparative study of two distinct nanofluids comprising identical nanoparticles but with different proportions. The two formulated nanofluids were composed of MWCNT (50%)/CuO (25%)/SiO_2_ (25%)/water and MWCNT (10%)/CuO (20%)/SiO_2_ (70%)/water. Their findings indicated that the quantity of nanoparticles exerts a profound influence on the thermal properties. Specifically, the first and second nanofluids exhibited 37.10 and 16.20% increases in thermal conductivity compared to the base fluid.

Among the mentioned nanoparticles, graphene and GO have gained a lot of attention from researchers because of their hydrophilicity, high thermal conductivity, high specific surface area, being more stable, requiring less pumping power, high mechanical strength, and low density.^31^ Banisharif et al.^32^ conducted an experiment with the primary objective of enhancing convective heat transfer within pipe heat exchangers utilizing water-based nanofluids. In their study, Cu, Fe_3_O_4_, MWCNT, and graphene nanoparticles (NGPs) were incorporated at varying volume fractions, ranging from 0.01 to 0.1%. Remarkably, at the highest nanoparticle concentration, notable improvements in thermal conductivity were observed, amounting to 2, 3, 2, and 5% for Cu, Fe_3_O_4_, MWCNT, and NPG nanofluids, consecutively. Furthermore, the heat transfer properties exhibited a significant improvement, reaching up to 20% when compared to both pure water and base fluids. In another study conducted by Fares et al.,^33^ graphene flakes were prepared using graphite foam, and the influence of nanofluids on convective heat transfer was investigated in a vertical shell and tube heat exchanger. A significant increase (29%) in the heat transfer coefficient was observed in the vertical configuration of the heat exchanger when graphene–water nanofluids were employed. Furthermore, the adoption of nanofluids led to enhanced thermal efficiency on both the hot and cold sides, resulting in enhancements of 24.4 and 7.3%, respectively. Demirkır et al.^34^ studied water-based nanofluids containing graphene platelets with varying particle mass fractions of 0.025, 0.1, and 0.2%. The evaluation of the mean heat transfer coefficient enhancements for the respective particle mass fractions revealed values of 7.3, 17.2, and 22.7%. Notably, the prepared nanofluids exhibited a remarkable maximum mean heat transfer coefficient enhancement of 36% at a Reynolds number of 3950 for a particle mass fraction of 0.2%. Barai et al.^35^ focused on synthesizing a novel nanocomposite, reduced graphene oxide-Fe_3_O_4_ (rGO-Fe_3_O_4_), and its corresponding nanofluid for investigating heat transfer properties. The thermal conductivity and convective heat transfer coefficients of the formulated rGO-Fe_3_O_4_ nanocomposite-based nanofluids represented significant enhancements of 83.44% and 845.4 W/m^2^ K, respectively, when evaluated at 0.2 vol % concentration and a temperature of 40 °C. Kanti and Maiya^36^ successfully synthesized nanoparticles consisting of GO and coal fly ash (CFA) to develop mono- and hybrid nanofluids (HNF) with a water base. Different particle mixture ratios of 50:50 and 30:70 and surfactant polyvinylpyrrolidone (PVP) were employed. The outcomes showed that the GO nanofluid indicated superior enhancements in thermal conductivity and viscosity compared to the investigated HNF. At a nanofluid concentration of 0.03 vol % and a temperature of 33 °C, the obtained thermal conductivity for the GO nanofluid was 1.046 W/m^2^ K. The performance enhancement ratio (PER) analysis indicated that both the GO nanofluid and HNF with a mixture ratio of 30:70 showcased favorable attributes for thermal applications across all temperatures. In contrast, HNF with a mixture ratio of 50:50 demonstrated thermal benefits only at temperatures of 45 °C.

Based on the extensive literature review on the effectiveness of nanoparticles, specifically GO nanoparticles, most studies have focused on measuring the heat transfer properties of considering different base fluids. Additionally, most research reported that adding nanoparticles could significantly improve the heat transfer features of the prepared nanofluids. However, none of the studies have considered the best composition of the considered base fluid in terms of better stability and lower temperature difference through the applied system. Furthermore, to the best of our knowledge, no previous study was conducted on a pilot scale with minimum errors to mimic real industry conditions. Thus, the findings of this comprehensive evaluation are reproducible in terms of industrialization feasibilities.

Herein, in this comprehensive assessment, an indirect heat exchanger was scaled down and constructed to correspond to an industrial rig. Initially, the best surfactant in the base fluid was chosen between industrial- and laboratory-grade ethylene glycol (EG). Subsequently, nanofluids with various GO nanoparticle contents were prepared using a base fluid of deionized water/ethylene glycol at a v/v% ratio of 30:70. Density, viscosity, specific heat capacity, thermal conductivity coefficient, Prandtl number, and Nusselt number were calculated by using provided sensors and obtained data. After optimization of the operating temperature and GO nanoparticle content, a new model for Nusselt number calculation was suggested.

## 2 Experimental Section

### 2.1 Design and Specifications of the Apparatus

**Figure 1:**
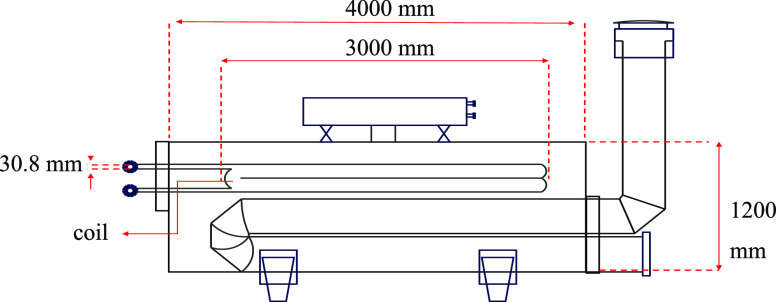
Schematic of an industrial-scale heat exchanger.

A laboratory heat exchanger apparatus was designed, considering the principles of similarity, to evaluate the critical factors influencing the heat transfer process, such as temperature variations between the gas inlet and outlet and the gas velocity passing through the coil. Precise pressure drop and temperature were obtained at both the coil’s inlet and outlet. The laboratory heat exchanger’s coil and shell were designed by scaling down the original dimensions of an indirect water bath heat exchanger with a volumetric flow rate of 2500 m^3^/h, as illustrated in Figure 1. The *L*/*D* ratio, representing the length-to-diameter ratio, was maintained, and a reduction scale of 1/10 was applied to achieve the desired size. Given the coil’s four-row back-and-forth configuration, the scaling down process was carried out in accordance with eq 1, wherein the subscript “a” denotes the parameters of the laboratory-scale apparatus and the subscript “I” denotes the parameters of the industrial-scale instrument. The resulting dimensions of the heat exchanger apparatus were calculated as 400, 152.4, 300, 9.525, and 8 mm for shell length, shell diameter, coil length, coil diameter, and coil pass account, respectively. The shell diameter was increased by one size to accommodate the expansion source. The shell was constructed using seamless steel, while the coil was made of copper oxide with a thickness of 0.8 mm and surface roughness (ε) equal to 0.0015 mm.
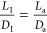
1

To achieve dynamic and kinematic similarity in the apparatus, two dimensionless parameters, namely, the Reynolds number (*Re*) and the Euler number (*Eu*), were employed. Instead of utilizing natural gas, compressed air was introduced into the system through a compressor and then directed through the coil. In adherence to the IGS standard of the Iran National Gas Company, the prescribed upper limit for the gas velocity passing through the coil (*V*) was 20 m/s, while the maximum allowable pressure drop (Δ*P*) across the coil was 1.75 bar. Accordingly, based on the thermodynamic properties of NG and air, the maximum admissible airflow velocity and allowable pressure drop within the coil were calculated consecutively using eqs 2 and 3, as presented in Table 1.^37,38^
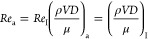
2
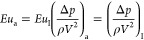
3

**Table 1:** Thermodynamic Properties of NG and Air

property	air	NG
density (ρ) (kg m^–3^)	1.2	0.654
viscosity (μ) (Pa s)	1.796 × 10^–5^	1.087 × 10^–5^
specific heat capacity (*C*_p_) (kJ kg^–1^ K^–1^)	1.007	2.254
thermal conductivity (λ) (W m^–1^ K^–1^)	0.025	0.033
maximum pressure (*P*) (bar)	74	1.75
maximum velocity (*V*) (m/s)	96	20

**Figure 2:**
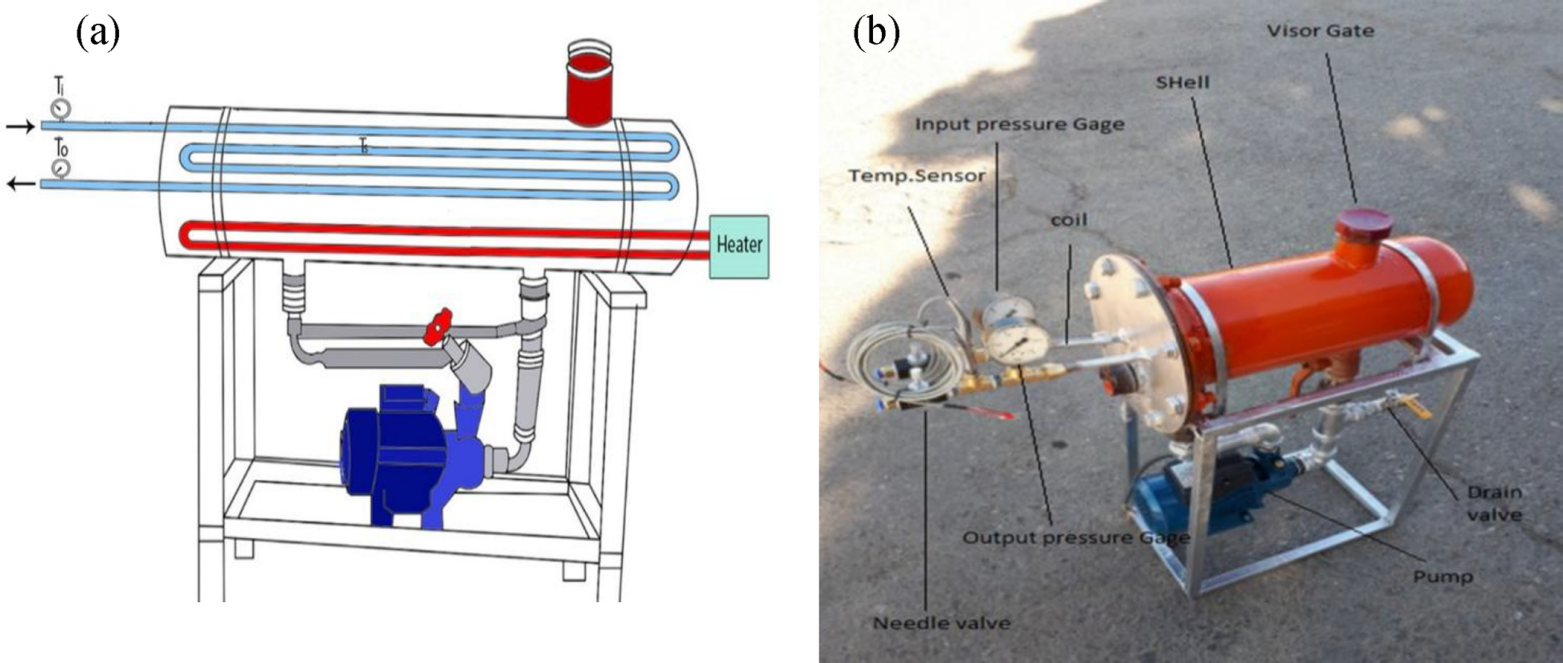
(a) Schematic and (b) picture of the apparatus.

The obtained values for the airflow velocity and Δ*P*, listed in Table 1, were derived from the anticipated maximum gas velocity and Δ*P* in the coil. Nevertheless, the acquired values did not accurately reflect the authentic conditions observed in the heat exchangers within the pressure reduction stations. For the aforementioned stations, it was ascertained that the maximum velocity and maximum pressure drop within the coil ranged from approximately 10 to 17 m/s and 10 mbar, respectively. As a result, the appropriate range of conditions encompassed an airflow velocity (*V*) between 10 and 17 m/s and a Δ*P* ranging from 0.45 to 0.5 bar. A control and monitoring system was implemented to achieve and maintain the specified conditions within the laboratory apparatus. The schematic of the constructed apparatus is illustrated in Figure 2.

The coil’s inlet and outlet pressures were measured using a Bourdon tube-type analog pressure, a gauge capable of quantifying pressure from 0 to 160 mbar. Temperature measurements at the coil’s inlet and outlet were carried out employing PT100 temperature sensors (uncertainty: ±0.1 °C). The temperature readings were acquired utilizing the digital module (Autonics-T4WM), with five sensors and measurement rates within the −200 to +400 °C range. To measure the surface temperature of the coil tube via the inspection valve, a digital temperature measuring device (TM-914c) was utilized with a temperature range spanning from −40 to +1200 °C. Three Ds18b20 sensors, featuring steel and stainless covers, were affixed to distinct points along the coil, thereby facilitating the temperature data collection. A needle valve, 0.25 in., was used to effectuate airflow regulation at both the inlet and outlet of the coil pipe. The circulation of the nanofluid within the shell was facilitated using a centrifugal pump with a power capacity of 0.5 hp to prevent the sedimentation of nanoparticles in the system. The nanofluid within the shell was heated using an element with a power rating of 1.5 kW, capable of providing a temperature range between 30 and 85 °C. Additionally, the element was equipped with a thermocouple that deactivated upon reaching the desired temperature. A dense air supply was provided using a 2 hp compressor, which generated pressure within the range of 8–9 bar. The pressure output was regulated through a pressure regulator, allowing for adjustments within the desired range of 30–35 psig.

### 2.2 Nanofluid Preparation

Ethylene glycol (EG), sodium dodecyl sulfate (SDS), and GO nanoparticles were purchased from Merck, Germany, Spectrum, US, and US Research Nanomaterials, respectively. An industrial grade EG (18154MF) was also provided by Fizogam Co., Iran. The characterizations of GO nanoparticles were validated using scanning electron microscopy (SEM, Zeiss, Germany), transmission electron microscopy (TEM, PHILIPS-CM120, Netherlands), and X-ray diffraction (XRD, PHILIPS-PW1730, Netherlands) techniques. In this study, a base fluid was prepared by combining water and EG in a 30:70 weight ratio. The base fluid’s stability was enhanced by using SDS as a surfactant. Initially, the surfactant was introduced into the base fluid (7.2 L) at a 1:1 weight ratio to GO nanoparticles. The mixture was subsequently stirred for 15 min using a magnetic stirrer. Subsequently, GO nanoparticles were added to the prepared base fluid considering diverse concentrations, 0.1, 0.05, and 0.01 wt %. The resulting mixture was subjected to dilution in an ultrasonic bath with a magnetic vibrator for 60 min, operating with a power input of 200 W and a frequency of 40 kHz.

### 2.3 Nanofluid Experiments and Calibration

Before the experimental tests proceed, it is imperative to calibrate the system. To achieve this, air was initially introduced into the heat exchanger coil, and the pressure and temperature at the inlet and outlet were measured while ensuring the closure of the coil outlet gauge. The objective was to ascertain that no temperature difference was observed under fixed pressure conditions, thereby affirming the proper functionality of the system. For reproducibility and minimizing errors within the entire system, the same calibration procedure was carried out using water, and this entire process was repeated nine times to attain steady-state condition. The inlet and outlet pressures were maintained at 1 and 0.5 bar throughout this calibration process, respectively. Once the calibration process was accomplished, the shell was filled with prepared nanofluids. Then, the nanofluid temperatures were set at 40, 55, 70, and 85 °C, while measurements were taken for the temperatures of the inlet and outlet air to the coil, the nanofluid, and the shell, as well as the inlet and outlet pressures.

## 3 Results and Discussion

### 3.1 Characterization of the GO Nanoparticle

**Figure 3:**
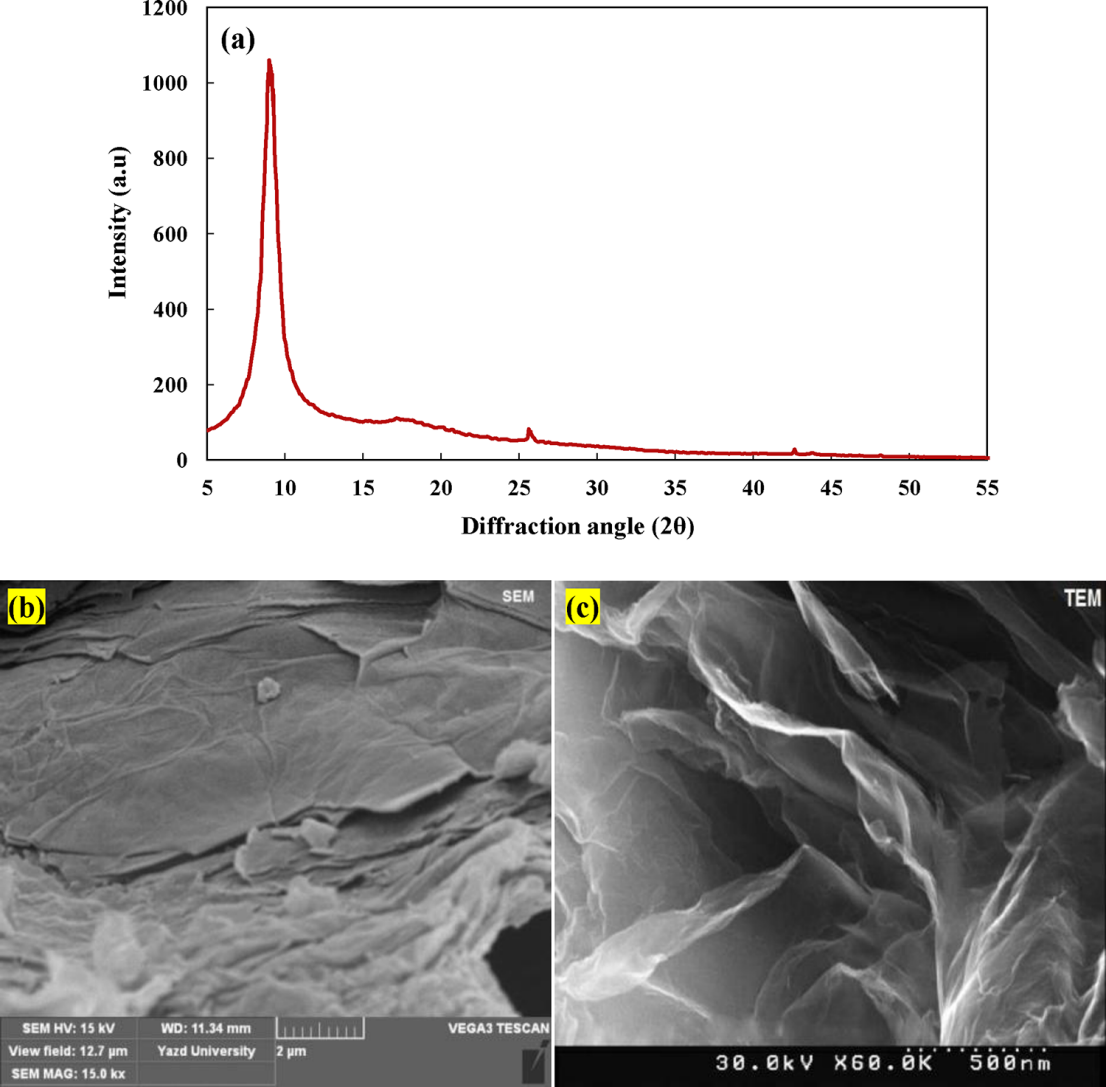
(a) XRD pattern, (b) SEM micrograph, and (c) TEM micrograph of the GO nanoparticle.

The GO nanoparticle was subjected to X-ray diffraction (XRD) analysis to confirm its purity (Figure 3a).^39,40^ The XRD pattern revealed a prominent peak at approximately 2θ = 10°, which is a characteristic feature indicative of graphene oxide (GO).^41^ No other prominent peaks were observed; therefore, there were no impurities. SEM micrographs (Figure 3b) provided a distinct view of the nanoparticle, indicating a multilayer structure. This characteristic is beneficial as it results in an enlarged surface area, which can be advantageous for various applications. Moreover, the TEM micrographs of the nanoparticles (Figure 3c) appeared semitransparent and had wrinkled-sheet structures, probably due to the presence of oxygen atoms.^42^

### 3.2 Base Fluid Selection

**Figure 4:**
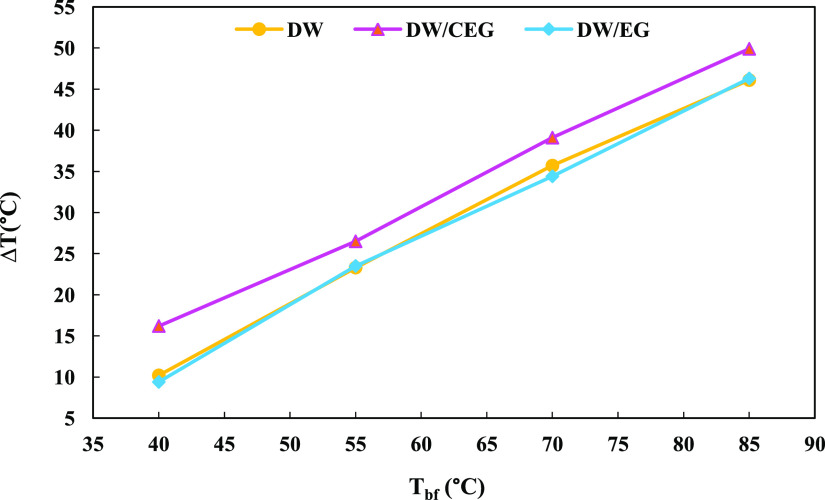
Temperature difference at various base fluid temperatures for DW, CEG, and DW/EG.

Initially, to compare and choose the efficient base fluid among deionized water (DW), commercial-grade EG (DW/CEG), and laboratory-grade EG (DW/EG), the inlet and outlet air temperature difference (Δ*T*) was recorded at four different temperatures for each base fluid (*T*_bf_). Considering the outcomes presented in Figure 4, DW/CEG and DW/EG demonstrated the most and least Δ*T*, respectively. As the thermal conductivity (λ) values of DW and EG (Merck) were 0.607 and 0.254 W m^–1^ K^–1^, respectively; hence, the obtained DW/EG with a weight ratio of 30:70 had a thermal conductivity equal to 0.456 W m^–1^ K^–1^. The higher λ for DW with regard to DW/EG can justify the recorded higher Δ*T* (nearly 4%) for DW. The distinctly higher Δ*T* for DW/CEG was due to the presence of polymer additives in the commercial EG. After the data were acquired, DW/EG was chosen as the base fluid for the rest of the study. The DW/EG properties considering the temperature (*T*, K) including viscosity (μ_bf_), specific heat (*C*_Pbf_), density (ρ_bf_), and thermal conductivity (λ_bf_) were determined through the application of specific equations (eqs 4–7),^10,43^ and the resulting data can be found in Table 2.
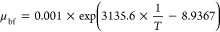
4
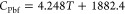
5
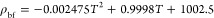
6
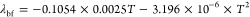
7

**Table 2:** Thermophysical Properties of DW/EG (30:70) at Varying Temperatures

*T*_bf_ (°C)	λ_bf_ (W m^–1^ K^–1^)	ρ_bf_ (kg m^–3^)	μ_bf_ (cp)	*C*_Pbf_ (J kg^–1^ K^–1^)	*T*_i_ (°C)	*T*_o_ (°C)	Δ*T*
40	0.364	1072.882	2.934	3212.661	21.1	30.5	9.4
55	0.371	1064.07	1.857	3276.381	22.2	45.7	23.5
70	0.376	1054.145	1.223	3340.101	22.7	57.1	34.4
85	0.380	1043.107	0.834	3403.821	23	69.3	46.3

### 3.3 Air Temperature Difference

**Figure 5:**
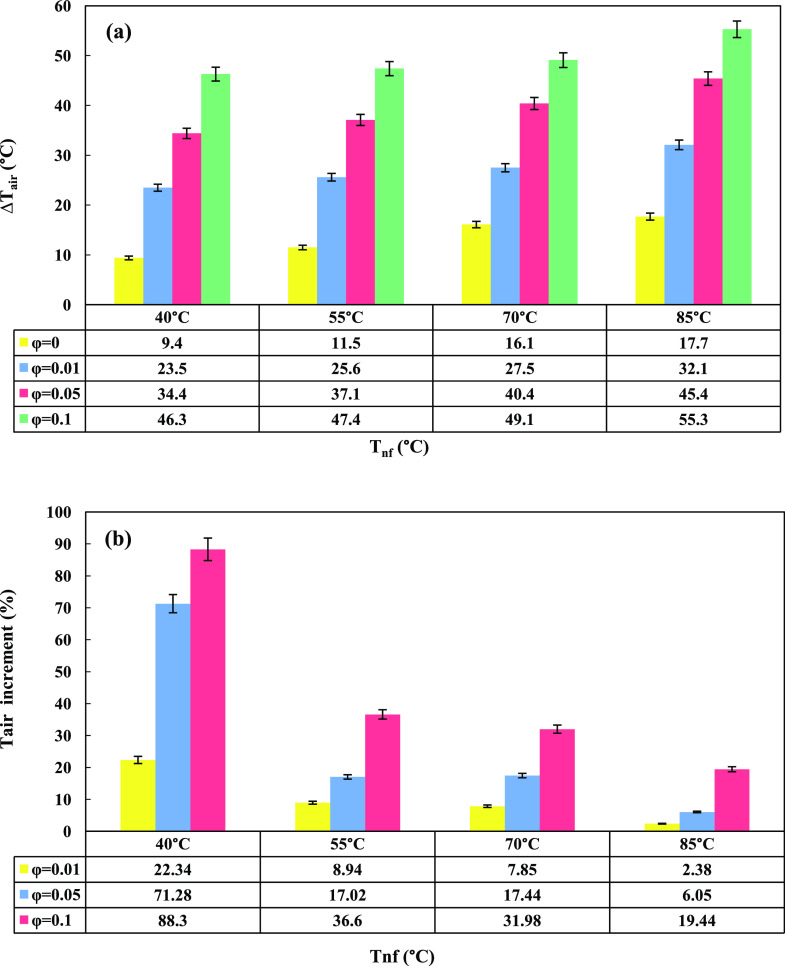
GO nanoparticle content and nanofluid temperature’s effect on (a) air temperature difference and (b) air temperature increment (%).

Nanofluids with GO nanoparticle contents of ϕ = 0.01, 0.05, and 0.1 wt % were prepared and tested using the designated apparatus, and the temperature difference of airflow (Δ*T*_air_) for each nanofluid was measured at different nanofluid temperatures (*T*_nf_ = 40, 55, 70, and 85 °C). The results for both nanofluids and the base fluid are depicted in Figure 5a. The least and most Δ*T*_air_ for each nanofluid was recorded at 40 and 85 °C, respectively. It was also concluded that by increasing the weight fraction of GO nanoparticles, Δ*T*_air_ increased. The observed phenomena can be explained by the exceptional characteristics of nanoparticles, including their high surface area, thermal conductivity, and mobility, which play a crucial role in enhancing heat transfer within nanofluids. Due to their small size, nanoparticles disperse uniformly in the fluid and remain suspended without settling, ensuring a stable mixture. The significant surface area enables strong interactions with the fluid molecules, resulting in improved thermal conduction. Additionally, the nanoparticles’ mobility through Brownian motion enhances convective heat transfer. As the nanofluid flows, the nanoparticles are carried along, intensifying their interaction with the fluid and promoting more efficient heat transfer. For a proper comparison of nanofluids with base fluid, the obtained data are reported as an increasing percentage of *T*_air_ in Figure 5b. The most enhancement was clearly for 0.1 wt % nanofluid at 40 °C.

### 3.4 Density

**Figure 6:**
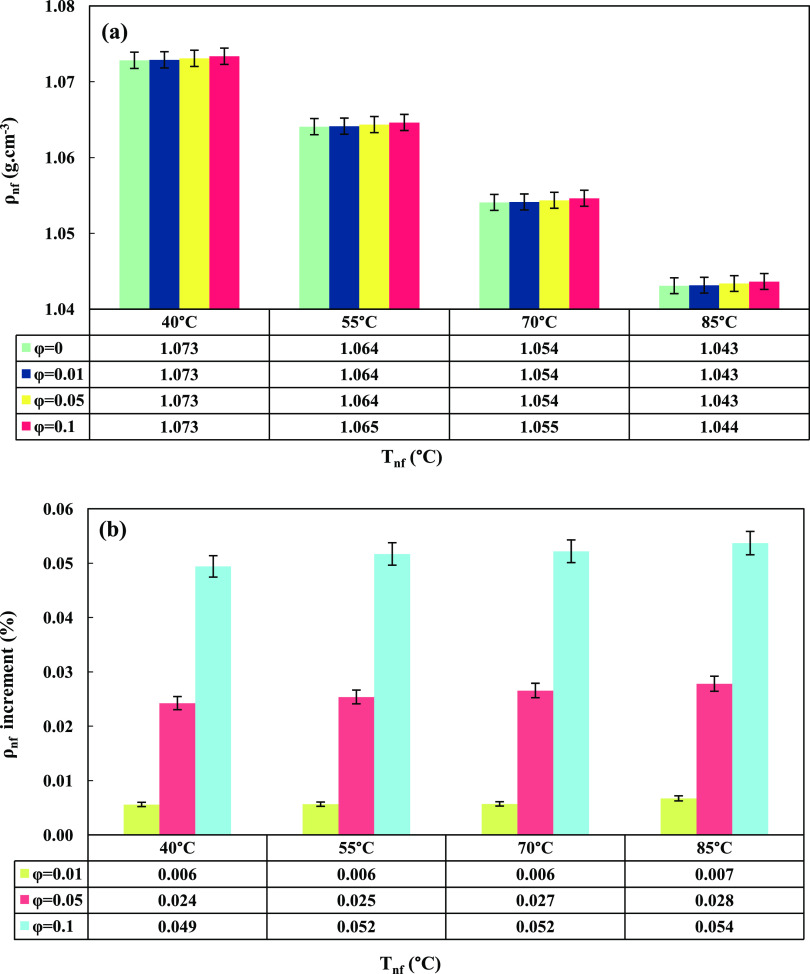
GO nanoparticle content and nanofluid temperature’s effect on (a) nanofluid density and (b) density increment (%).

Figure 6a demonstrates the effect of GO nanoparticle contents on the nanofluid’s density at various temperatures. The densities of nanofluids (ρ_nf_) and base fluid (ρ_bf_) were calculated using eq 8 (known as the Pak and Cho equation) and eq 6, respectively. Furthermore, for a better understanding of the nanofluid temperature (*T*_nf_) effect and GO nanoparticle contents (ϕ) on the density, the density increment (%) is exhibited in Figure 6b. The calculations were based on the volume fraction of nanoparticles, which was calculated using eq 9, where ϕ_v_, ϕ, ρ_p_, and ρ_bf_ represent v/v%, wt %, density of nanoparticle, and density of base fluid, respectively.^43^
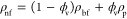
8
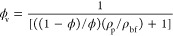
9

As evident from Figure 6a, ρ_nf_ decreased with an increase in *T*_nf_ and increased with the addition of ϕ. The maximum ρ_nf_ was observed at the lowest temperature of 40 °C and ϕ = 0.1 wt %. Additionally, referring to Figure 6b, the nanofluid’s density increment exhibited minimal variations and remained nearly constant with increasing ϕ. The increase in the nanofluid density with a higher nanoparticle content can be attributed to the higher density of the nanoparticles themselves. As the concentration of nanoparticles increases, their contribution to the overall mass of the nanofluid becomes more significant, leading to a corresponding increase in the nanofluid’s density. Conversely, the decrease in nanofluid density with a rise in temperature is a consequence of thermal expansion. With increased temperature, the kinetic energy of the fluid molecules intensifies, causing them to experience greater molecular motion and separation. This phenomenon results in an expansion of the fluid’s volume, leading to a reduction in its density. It is worth noting that the density change in nanofluids due to temperature is generally smaller compared to that in pure liquids because the presence of nanoparticles tends to hinder the extent of thermal expansion. Nevertheless, the thermal expansion effect can still cause a measurable decrease in the density with increasing temperature.

### 3.5 Specific Heat Capacity

**Figure 7:**
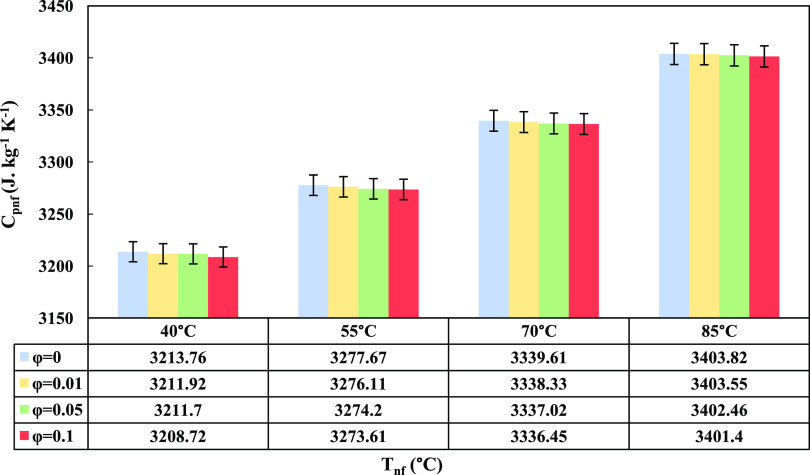
GO nanoparticle content and nanofluid temperature’s effect on the nanofluid’s specific heat capacity.

One of the important thermophysical properties of a nanofluid is specific heat capacity (*C*_Pnf_), which was obtained according to eq 10.^43^Figure 7 represents the effect of GO nanoparticle contents at different temperatures on the specific heat capacity considering the specific heat capacity of nanoparticles (*C*_Pp_).
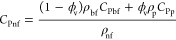
10

Based on the data shown in Figure 7, the specific heat capacity of the nanofluid increased with temperature and decreased with a higher content of GO nanoparticles. The highest *C*_Pnf_ was seen at a temperature of 40 °C and ϕ = 0.01 wt %. The nanofluid tends to exhibit higher specific heat capacity at lower concentrations of nanoparticles and lower temperatures due to the combined effects of nanoparticle–fluid interactions and thermal behavior. At lower concentrations of nanoparticles, there is less interaction between the nanoparticles and fluid molecules. This allows the fluid to retain its intrinsic specific heat capacity, typically higher than that of the nanoparticles. In contrast, at higher nanoparticle concentrations, the presence of a larger number of nanoparticles hinders the fluid’s ability to absorb and store heat efficiently, leading to a reduction in the overall specific heat capacity of the nanofluid. At lower temperatures, the thermal energy of the fluid and nanoparticles is relatively low. In this regime, the fluid molecules exhibit less vigorous motion, allowing them to retain a greater portion of the supplied heat energy and, consequently, higher specific heat capacity. However, at higher temperatures, the increased kinetic energy of the fluid molecules leads to more extensive thermal agitation, reducing the fluid’s ability to store heat energy effectively and resulting in a decline in specific heat capacity.

### 3.6 Dynamic Viscosity

**Figure 8:**
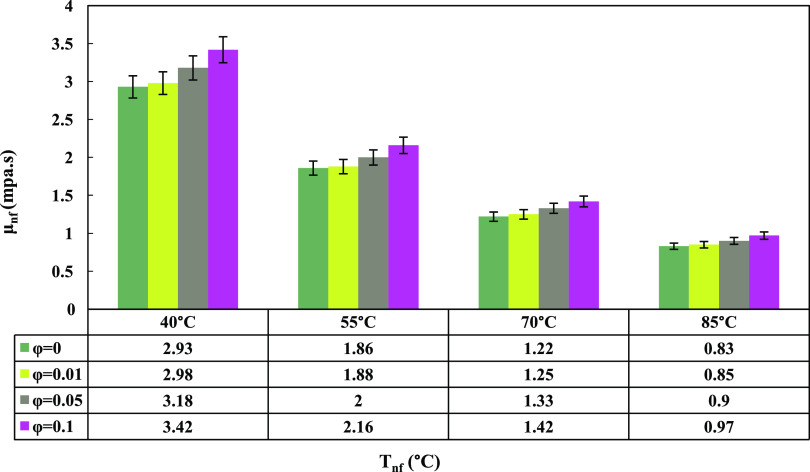
GO nanoparticle content and nanofluid temperature’s effect on nanofluid viscosity.

The viscosity of nanofluids (μ_nf_) was also acquired, and it is presented in Figure 8 and Table 3 using the Brinkman equation (eq 11) based on the viscosity of base fluid for different contents of GO nanoparticles at various temperatures.^44^
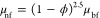
11

**Table 3:** Ratio of Nanofluid Viscosity to Base Fluid Viscosity

μ_nf_/μ_bf_ at	ϕ = 0.01 wt %	ϕ = 0.05 wt %	ϕ = 0.1 wt %
*T*_nf_ = 40 °C	1.017	1.085	1.167
*T*_nf_ = 55 °C	1.011	1.075	1.161
*T*_nf_ = 70 °C	1.025	1.090	1.164
*T*_nf_ = 85 °C	1.024	1.084	1.169
average	1.019	1.084	1.165

According to the diagram, an increase in the concentration of the GO nanofluid (ϕ) increased the dynamic viscosity. This phenomenon was attributed to the introduction of GO nanoparticles into the base fluid, which raised interactions between the nanoparticles and the base fluid molecules. As the quantity of nanoparticles in a fixed amount of the base fluid increased, the van der Waals forces among the GO nanoparticles strengthened, resulting in the formation of larger nanoparticle clusters. These clusters hindered the smooth movement of the water and EG layers over each other, ultimately causing an increase in the overall viscosity of the nanofluid. Furthermore, as the nanofluid temperature increased, it caused an increase in the Brownian motion of the nanoparticles, overcoming the van der Waals forces. Consequently, the viscosity of the nanofluid decreased. Thus, the minimum μ_nf_ value corresponded to a temperature of 85 °C and a concentration of ϕ = 0.01 wt %. In Table 3, the ratio of nanofluid viscosity to base fluid viscosity displayed an ascending trend with increasing weight concentration of the nanofluid. This trend demonstrated the growing influence of GO nanoparticle concentration on the nanofluid’s viscosity.

### 3.7 Thermal Conductivity Coefficient

One of the influential properties of a fluid in heat transfer processes, involving both conduction and convection, is its thermal conductivity. The high thermal conductivity signifies an elevated heat transfer rate through each of the two mentioned mechanisms. Consequently, the thermal conductivity of nanofluid (λ_nf_) was calculated using the Yu and Choi equation (eq 12) in which λ_P_ and λ_bf_ represent the thermal conductivity of GO nanoparticles and base fluid, respectively.^20^
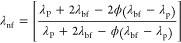
12

**Figure 9:**
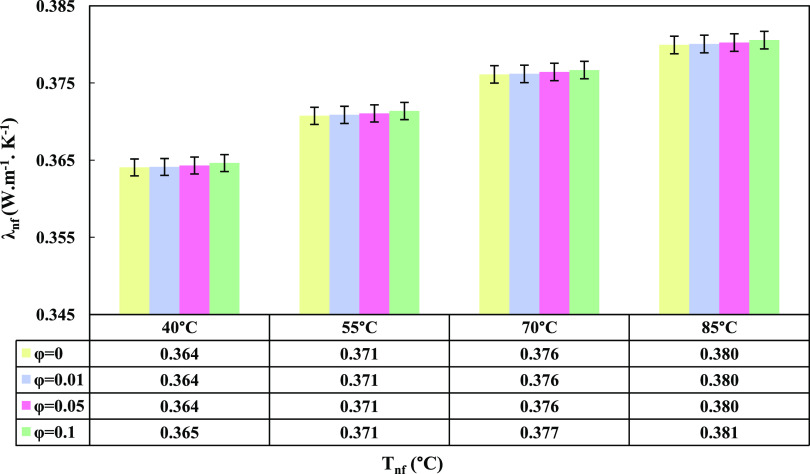
GO nanoparticle content and nanofluid temperature’s effect on nanofluid thermal conductivity.

Based on the acquired plotted thermal conductivity outcomes in Figure 9, λ_nf_ increased with the addition of temperature and concentration. In fact, at a temperature of 85 °C and ϕ = 0.1 wt %, this parameter reached its highest value and exhibited an increase of more than 0.15%. Water and EG are among the commonly utilized coolants in heat exchangers; however, they possess relatively low thermal conductivities compared to solids. By incorporating nanoparticles into the base fluid, the thermal conductivity of the base fluid can be increased. The main reason for the increase in the thermal conductivity in nanofluids is 2-fold. First, an increase in temperature leads to an elevation in the Brownian motion of suspended nanoparticles in the nanofluid. This random motion results from the stochastic collisions of molecules or particles in the fluid and aids in the heat transfer process from warmer regions to cooler regions. Second, the augmentation in the content of nanoparticles within the nanofluid leads to an increase in the surface-to-volume ratio of the fluid. Consequently, a higher number of particle collisions occur, further enhancing the overall heat transfer within the nanofluid.

### 3.8 Prandtl and Nusselt Numbers

One of the important dimensionless numbers in the analysis of convective heat transfer is the Prandtl number (*Pr*_nf_), which is defined for nanofluids as given in eq 13(45):
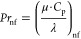
13

**Figure 10:**
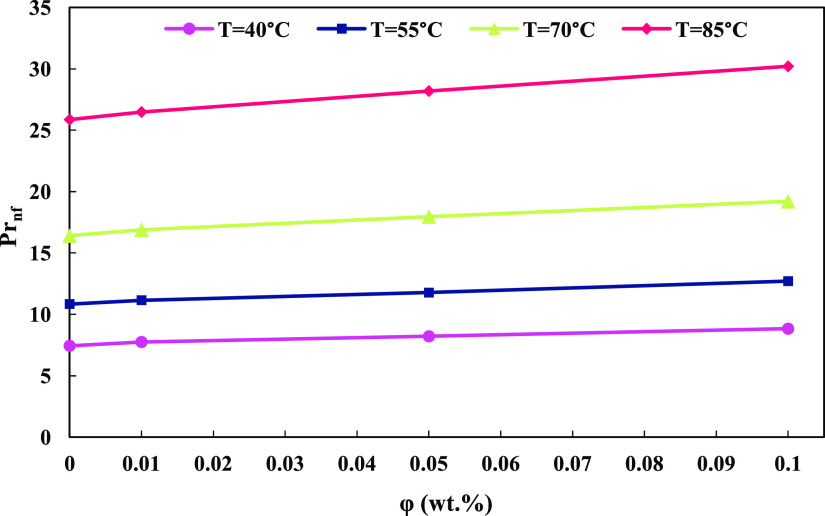
GO nanoparticle content and nanofluid temperature’s effect on Prandtl number.

As the Prandtl number decreases, the fluid’s capability to conduct heat transfer increases, while a larger *Pr*_nf_ indicates a higher potential for convective heat transfer. The variations of the Prandtl number for nanofluids at different temperatures and weight concentrations are shown in Figure 10. As observed in the graph, with an increase in temperature, *Pr*_nf_ decreased, and with an increase in ϕ of the nanofluid, the Prandtl number increased. Based on the defined relationship and the trend displayed in the graph, it can be concluded that with an increase in temperature, the nanofluid’s capability transitions from a convective heat transfer mechanism to a conductive heat transfer mechanism.

Another dimensionless parameter that plays an essential role in nanofluid thermal activities is the Nusselt number (*Nu*) because it quantifies the enhancement in heat transfer resulting from the presence of nanoparticles in the base fluid. For the experimental calculation of *Nu*, the convective heat transfer coefficient of air (*H*) was initially obtained using eqs 14–16 and then put into eq 17.^37^ The thermophysical properties of air were determined based on the average inlet and outlet temperatures of the heater. *ṁ* represents the mass flow rate of air, *C*_p_ is the specific heat of air, and *T*_i_ and *T*_o_ are the inlet and outlet temperatures of the air coil, respectively. Additionally, *A* represents the heat transfer surface area of the coil, *D* is the pipe’s outer diameter, *T*_s_ is the surface temperature of the coil, and *T*_f_ is the average of the inlet and outlet temperature of air.
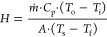
14
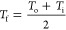
15
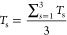
16
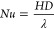
17

The Dittus–Boelter equation, which is shown in eq 18, can be used to calculate the Nusselt number.^46^ If the fluid is cooling, the value of *n* is 0.33, whereas if the fluid is heating, the value of *n* is 0.4; hence, the *n* was set to 0.4.
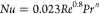
18

**Figure 11:**
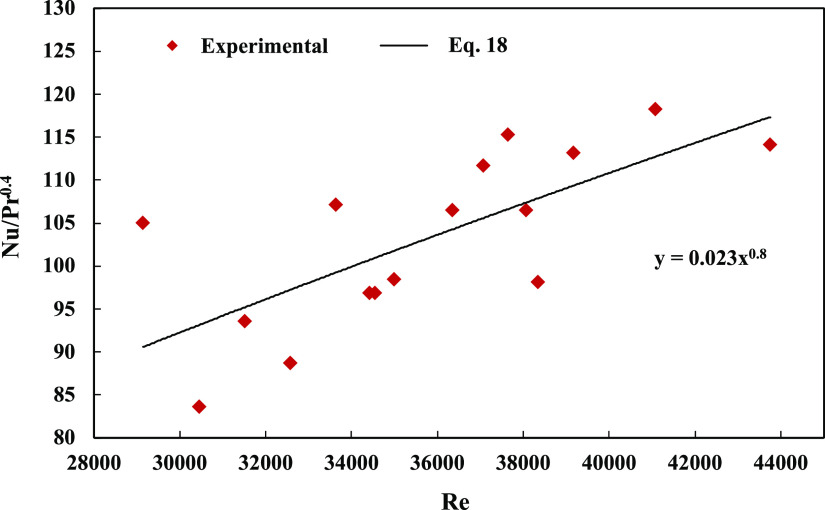
Relation between Reynolds, Prandtl, and Nusselt numbers using the experimental method and Dittus–Boelter equation.

Subsequently, to calculate  in eq 18, the Prandtl number of air was set to be constant at *Pr* = 0.72 over a wide range of Reynolds numbers, aiming to ensure its stability. Additionally, the Dittus–Boelter equation was employed to depict the relationship between the Nusselt and Reynolds numbers, as illustrated in Figure 11, where the maximum error difference between these equations was approximately 18%.

In this work, we have successfully derived a formula to calculate the average Nusselt number as a function of the Prandtl number, mass fraction, Peclet Number (*Pe*), and Reynolds number based on our experimental results. To achieve this formula, we utilized a data set and employed Excel programming to calculate the constant values. By employing the method of least-squares regression on the data set points, we obtained correlation values, shown in eq 19:
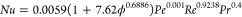
19

This developed equation holds true for Prandtl numbers ranging from 0.7 to 50. Notably, the average deviation of this equation from our experimental data is approximately 13%. This newly derived correlation provides a valuable tool for predicting the Nusselt number in various heat transfer scenarios involving nanofluids with different Prandtl numbers, mass fractions, and Reynolds numbers.

## 4 Uncertainty Analysis

Two of the important aspects to consider in laboratory experiments are the evaluation of the accuracy of obtained results and the determination of the percentage of error. The relationship used for error determination is represented as eq 20(10):
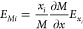
20

In the equation, the parameter *x*_*i*_ is a measurable quantity, *M* is the calculated quantity based on the measured parameter,  denotes the measurement error, which is given by (minimum measurable quantity)/(measurement precision), and *E*_*Mi*_ represents the maximum possible error in measuring a quantity. Thus, the maximum error of parameter *M* is calculated by combining the errors of each individual parameter *x*_*i*_ using the following formula:
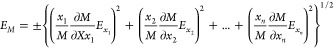
21

The calculated uncertainties and maximum errors in measurements were all calculated, and they are presented in Table 4.

**Table 4:** Maximum Measured Errors

parameter	error (%)
pipe diameter	±1.05
pipe length	±3.33 × 10^–3^
inlet temperature (*T*_i_)	±5.12 × 10^–1^
outlet temperature (*T*_o_)	±3.27 × 10^–1^
Δ*T* = *T*_o_ – *T*_i_	±0.9
average temperature of the pipe wall	±0.357
inlet pressure	±4.5
outlet pressure	±4.5
nanofluid temperature	±0.25
effective heat transfer surface area of the coil	±1.05
mass flow rate	±1.07
overall heat transfer coefficient	±3.76
Nusselt number calculation	±3.9
thermal resistance	±3.12

## 5 Technological Constraints, Environmental Concerns, and Scalability Prospects

### 5.1 Technological Constraints

Manufacturability of GO nanofluids on a large scale presents challenges due to the need for precise control over nanoparticle dispersion and stability. The synthesis methods, such as sonochemical or hydrothermal routes, must be scalable while ensuring a consistent quality and cost-effectiveness. Compatibility with existing heat exchanger materials is crucial to preventing corrosion or fouling issues. Techniques such as coating or material modifications may be necessary to enhance compatibility and durability. Integrating GO nanofluids into industrial heat exchangers requires a thorough understanding of flow dynamics, pressure drops, and thermal performance. Computational fluid dynamics (CFD) simulations can help optimize designs for efficient heat transfer, but real-world implementations may require further refinement.

### 5.2 Environmental Concerns of Materials Use

The environmental impact of GO nanofluids includes potential risks if nanoparticles are released into the ecosystems. Proper containment measures and waste management protocols are essential to minimize the environmental impact. Additionally, the production of GO nanofluids involves resource-intensive processes such as graphite precursor synthesis and nanoparticle functionalization. Sustainable sourcing practices and recycling efforts can reduce resource consumption and waste generation. Life cycle assessments (LCAs) indicate potential environmental benefits in terms of energy savings and reduced emissions compared with traditional heat transfer fluids. However, careful consideration of end-of-life disposal is crucial to avoid pollution and ensure sustainability.

### 5.3 Scalability Prospects and Large-Scale Applications

As production techniques for GO nanofluids mature and demand increases, the cost is expected to decrease, making them more economically viable for industrial adoption. Bulk purchasing and optimized manufacturing processes can further enhance the cost-effectiveness. Beyond heat exchangers, GO nanofluids show promise in enhancing the efficiency of various industrial processes, such as cooling systems, energy storage devices, and electronics cooling. Collaborations with industries focusing on nanomaterial applications can drive innovation and cross-sectoral advancements. Market acceptance hinges on demonstrating performance benefits, safety profiles, and regulatory compliance. Standardization efforts, certification programs, and stakeholder collaborations are vital for navigating regulatory challenges and fostering market growth.^47−49^

## 6 Conclusions and Future Research Considerations

The main findings of this research, based on the acquired results and the use of graphene oxide nanofluid in the laboratory-designed heater, are as follows:

1. The maximum temperature difference reported between the inlet and outlet air was 88% at a weight concentration of 0.1% and a temperature of 40 °C.
2. An increase in the nanofluid temperature led to a decrease in its density, while an increase in the weight concentration percentage of the nanofluid increased its density. The maximum density corresponded to the lowest temperature of 40 °C and a weight concentration of 0.1 wt %.
3. The viscosity decreased with an increase in the nanofluid temperature, whereas an increase in the weight concentration percentage resulted in a rise in the nanofluid viscosity. Hence, the maximum viscosity was associated with the highest weight concentration and the lowest temperature.
4. The specific heat capacity rose with an increase in temperature and declined with an increase in the weight concentration percentage. However, this decrease was relatively negligible.
5. As the temperature increased, the Prandtl number decreased, while an increase in the weight concentration of the nanofluid resulted in an elevation of the Prandtl number. The highest Prandtl number was achieved at 40 °C with a weight concentration of 0.1 wt %.
6. The difference between the theoretically calculated Nusselt number and the experimentally obtained one was nearly 18%.

The results indicated that considering the operational pressure in natural gas pressure reduction stations, the use of graphene oxide nanofluid is suitable and satisfactory for creating a temperature difference of 20–30 °C at the outlet of indirect water heaters.

In the conducted research, significant efforts were made to ensure precision throughout all experimental stages under various laboratory and atmospheric conditions. Multiple repetitions of the experiments were carried out to obtain results with minimal error. However, it should be noted that employing nanofluids in indirect water heaters at pressure reduction stations is the subject of both theoretical and experimental investigations. To effectively implement nanofluids in indirect water heat exchangers, the following aspects need to be considered:

- Specific economic analysis to justify the use of nanofluids in indirect water heaters, especially due to their larger volume.
- Investigation of the corrosion of metal surfaces caused by nanofluids and the implementation of cathodic protection techniques to mitigate potential issues.
- Modeling the process of utilizing nanofluids in indirect water heaters and understanding their impact on the natural gas system.

## Data Availability

All data generated or analyzed during this study are included in this published article.

## Nomenclature

*A*: surface area (m^2^)
*L*: length (m)
*D*: outer diameter (m)
*Re*: Reynolds number
*Eu*: Euler number
*P*: pressure (bar)
*V*: maximum velocity (m/s)
*T*: temperature (°C)
*C*_p_: specific heat capacity (J kg^–1^ K^–1^)
*Nu*: Nusselt number
*Pe*: Peclet number
*H*: convective heat transfer coefficient (W m^–2^ K^–1^)
*Pr*: Prandtl number
*X*_*i*_: measurable quantity
*M*: calculated quantity
measurement error
*E*_*Mi*_: maximum possible error in measuring a quantity
**Greek Letters**
*ṁ*: mass flow (kg s^–1^)
ρ: density (kg m^–3^)
μ: dynamic viscosity (Pa s = kg m^–2^ s^–1^)
λ: coefficient of thermal conductivity (W m^–1^ K^–1^)
ϕ: weight fraction (wt %)
ϕ_v_: volume fraction (v/v%)
ε: surface roughness (mm)
**Subscripts**
bf: base fluid
nf: nanofluid
i: inlet
o: outlet
a: laboratory-scale apparatus
I: industry-scale apparatus
p: nanoparticle
s: surface of coil
